# Neuronal plasticity during sleep slow wave oscillations

**DOI:** 10.1186/1471-2202-15-S1-P216

**Published:** 2014-07-21

**Authors:** Yina Wei, Giri P Krishnan, Maxim Bazhenov

**Affiliations:** 1 Department of Cell Biology and Neuroscience, University of California Riverside, CA 92521 USA

## 

Sleep is critical for regulation of synaptic efficacy, consolidation of memories and learning. It has been proposed that synaptic plasticity associated with sleep rhythms could contribute to consolidation of memories acquired during wakefulness. It was suggested that in the active state of sleep slow wave oscillation, the hippocampal formation activates latent memories stored in the neocortex (replay) and induce permanent changes in synaptic conductances.

In this study, we present a thalamocortical network model of the slow-wave sleep activity characterized by repeatable (< 1 Hz) transitions between active (Up) and silent (Down) states of the network. The model consisted a layer of thalamic relay (TC) and reticular (RE) neurons in the thalamus as well as a model of cortical column with pyramidal neurons and inhibitory interneurons. All neurons were modeled based on the Hodgkin-Huxley kinetics. Spike-timing dependent synaptic plasticity was implemented to regulate synaptic efficacy. The slow oscillation was driven by intracortical dynamics; active states were initiated by the spontaneous miniature synaptic releases at different network sites [1] and propagated within the cortical network. The properties of slow oscillations depended on the intrinsic conductances, miniature synaptic releases, and the synaptic strength. In agreement with previous experimental studies, transition from the active to the silent state occurred more synchronously than the active state initiation.

We found that the pattern of the active state propagation depended on the spatiotemporal pattern of activity on the previous cycle of slow oscillation and could be influenced by external input. Because of the refractoriness properties of the network, a probability of the next active state initiation was higher for the network site that initiated activity at the previous cycle. Furthermore, even weak external stimulation delivered to the network results in increase probability of up-state induction at the stimulation location. This suggests that spatially and temporally sparse hippocampal input could influence the spatiotemporal pattern of slow oscillation.

Location of the initiation site and the pattern of active state propagation determined the relative timing of spiking in cortical neurons. When spike-time dependent synaptic plasticity (STDP) was implemented, there was a net decrease in synaptic strengths and, at the same time, an increase in the strength of specific synapses which were associated with the sequence replay. The change in synaptic weights between any two neurons was determined by the direction of active state propagation and by the distance between the neurons.

Our study propose a mechanism of how interaction between cortically generated slow waves and sparse external input, possibly representing input from hippocampal formation, may lead to reorganization of synaptic strength during stage 3/4 sleep.

## References

[B1] Timofeev I Grenier F Bazhenov M Sejnowski TJ Steriade M Origin of slow cortical oscillations in the deafferented cortical slab Cerebral Cortex 2000 15 1185 1199 10.1093/cercor/10.12.1185 11073868

